# GeneFriends: a human RNA-seq-based gene and transcript co-expression database

**DOI:** 10.1093/nar/gku1042

**Published:** 2014-10-31

**Authors:** Sipko van Dam, Thomas Craig, João Pedro de Magalhães

**Affiliations:** Integrative Genomics of Ageing Group, Institute of Integrative Biology, University of Liverpool, Liverpool, UK

## Abstract

Co-expression networks have proven effective at assigning putative functions to genes based on the functional annotation of their co-expressed partners, in candidate gene prioritization studies and in improving our understanding of regulatory networks. The growing number of genome resequencing efforts and genome-wide association studies often identify loci containing novel genes and there is a need to infer their functions and interaction partners. To facilitate this we have expanded GeneFriends, an online database that allows users to identify co-expressed genes with one or more user-defined genes. This expansion entails an RNA-seq-based co-expression map that includes genes and transcripts that are not present in the microarray-based co-expression maps, including over 10 000 non-coding RNAs. The results users obtain from GeneFriends include a co-expression network as well as a summary of the functional enrichment among the co-expressed genes. Novel insights can be gathered from this database for different splice variants and ncRNAs, such as microRNAs and lincRNAs. Furthermore, our updated tool allows candidate transcripts to be linked to diseases and processes using a guilt-by-association approach. GeneFriends is freely available from http://www.GeneFriends.org and can be used to quickly identify and rank candidate targets relevant to the process or disease under study.

## INTRODUCTION

The rapid expansion of microarray data over the past decade has resulted in large repositories which have been employed in various meta-analyses. This has led to a better understanding of many biological processes and the identification of gene functions, biomarkers and targets for several diseases (1–3). Co-expression is a type of meta-analysis, which describes the expression of genes relative to each other and has been used for over a decade (4). This method has proven effective at assigning putative functions to genes based on the functional annotations of the genes they are co-expressed with, as well as better understand the underlying regulatory networks (5–8). Examples of tools utilizing co-expression data derived from public databases are GeneFriends (see below), COXPRESdb, CORNET, mouseMap, Genevestigator and STARNET2 (9–15). All of these works have used microarray data to construct co-expression networks, albeit using different metrics and approaches. Co-expression analyses have identified novel genes to be involved in diseases such as cancer (8,16), schizophrenia (17) and type 2 diabetes (18), or processes such as stem cell regulation (19) and the cell cycle (20).

**Figure 1.**
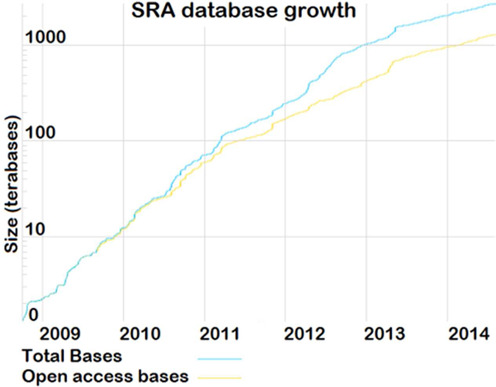
Exponential growth curve (log scale) of RNA-seq data taken from the Short Read Archive (SRA) (25).

Transcriptome sequencing (RNA-seq) is a powerful and emerging technology that allows researchers to measure differential expression of genes more accurately than when using microarrays (21). Like microarray databases, RNA-seq databases are growing exponentially (Figure 1) (22), creating the opportunity for meta-analyses similar to those conducted using microarrays, such as co-expression analysis. RNA-seq also measures expression of different splice variants and non-coding RNAs (ncRNAs), which can play important roles in gene expression regulation (23,24). The approximately 20 000 human genes only make up a small portion of the over 60 000 coding and non-coding RNAs (25) that encode the over 200 000 transcripts measured using RNA-seq (26), which greatly increases the challenges faced by researchers when interpreting RNA-seq results. A bottleneck in RNA-seq analyses is that even though a large number of transcripts can be detected as differentially expressed, often many have not been well studied. It is frequently unclear what possible functions poorly studied genes, specially non-coding ones, may have. As such, interpreting results from RNA-seq experiments and understanding the mechanisms involved in the disease or process under study is often impeded. Given the growing community of researchers employing RNA-seq, there is an unmet need for resources that help interpret results from such experiments. Moreover, the growing number of genome resequencing efforts and genome-wide association studies often associate loci containing poorly studied genes, such as ncRNAs, with diseases and traits (27,28), and there is a need to infer putative functions and interaction partners of new candidate genes (27,29).

Given the rapidly evolving sequencing technologies, there are now more RNA-seq samples available than there were microarrays at the time of the construction of the first widely used plant (30) and mammalian (31) co-expression websites. Recently the first co-expression analysis using RNA-seq data was conducted using 21 striatal samples and showed that co-expression networks created from RNA-seq data are more robust than those created from microarray data (32). This co-expression map, however, is striatal-specific and is not available online to the research community. No RNA-seq-based co-expression database is currently available for humans or for biomedical models (co-expression tools like CORNET, Genevestigator and COXPRESdb are based on microarray data). In this work, we developed the first online RNA-seq co-expression database for the bioscience community.

We had previously created an online co-expression analysis platform using over 3000 microarray data sets to facilitate the identification of candidate gene targets based on a user-defined list of disease- or process-related genes (13). This tool, entitled GeneFriends, can be used to assign putative functions to poorly studied genes using a guilt-by-association method (i.e. by investigating which genes a given poorly-studied gene is co-expressed with); it can also identify and prioritize novel candidate genes for further study based on a seed list of genes associated with a given disease or process, effectively allowing researchers to identify novel genes related to their study without the need for conducting a microarray or RNA-seq experiment. This tool has been successfully used to identify novel cancer-related genes that were validated experimentally (13). Whilst many tools are available to identify the function of genes and associate new genes with a seed list based on different interaction data (15,33–35) information on interaction of non-coding RNAs is more limited. Therefore in this work we have created and integrated into GeneFriends a co-expression map constructed from RNA-seq data, which allows for a better understanding of the regulatory patterns of ncRNAs in relation to mRNAs. Since RNA-seq allows researchers to assess the expression of different transcripts rather than only the gene level expression, we have also constructed a transcript co-expression map. This is particularly of interest since different transcripts originating from the same gene can have different functions (36) and co-expression is an easy way to detect different expression partners, suggesting different functionality.

Understanding the regulated and coordinated changes that occur between ncRNA and coding (inc. splice variants) expression may reveal novel important players in biological processes and diseases. Furthermore, RNA-seq has a larger dynamic range and measures expression of more genes including those previously un-annotated. These include ncRNAs such as microRNAs and long intergenic non-coding RNAs (lincRNAs), which may be crucial in understanding the mechanisms behind disease and biological pathways. This co-expression map allows these RNAs to be associated with known genes for inferring their function as well as with diseases, processes and pathways, leading to new associations that can be further investigated experimentally. GeneFriends is freely available on http://www.genefriends.org.

## CONSTRUCTION OF THE RNA-SEQ-BASED CO-EXPRESSION MAP

The RNA-seq-based addition to GeneFriends represents two co-expression maps: one containing genes (both coding and non-coding) and the other containing transcripts. The RNA-seq-based co-expression map was constructed using 4133 quality-controlled RNA-seq samples across 240 studies obtained from the SRA database (37) (Supplements 1). Our aim is to create a co-expression map that defines the behavior of genes under different circumstances (Supplements 1). For condition-specific genes a co-expression map created from a smaller set of samples may result in a more accurate result (14,38), but this is not the purpose of this tool, which is aimed at identifying the general role and associations of genes and transcripts.

Each sample complied with the following criteria:

1. Measured using the Illumina HiSeq2000 platform (although in future updates we anticipate also incorporating more recent platforms, like HiSeq2500)
2. Contained at least 10 million reads
3. Used a cDNA library preparation protocol
4. A minimum of 60% of the reads mapped to the Ensembl GRCh37 human genome (25)

The samples were mapped using STAR (39) and read counts per gene were determined by a custom Java program named ReadCounter. We opted to create our own counting tool since the widely used HTseq tool (40) was too slow for our purposes. ReadCounter is more efficient running approximately 3-fold faster on a single core (not shown). Additionally ReadCounter utilizes multithreaded technology which, using eight cores on our system, resulted in a 15–20-fold faster runtime. For benchmarking, ReadCounter has extra options that allow results to be identical to those obtained from HTseq, albeit at a much faster rate. Moreover, ReadCounter can more accurately assess the gene of origin in case multiple genes are overlapping on the genome, utilizing the overlap size of the reads with the different genes in a certain region. This advantage has been utilized when constructing our co-expression map. Furthermore, ReadCounter has another advantage of automatically counting the number of reads mapping to introns as well as reporting ambiguously mapping genes in a separate column. ReadCounter is written in Java and can be run using a command line in the terminal or command prompt (Mac/Linux/Windows) without the requirement for installation. The tool is free to use and publicly available at http://www.genefriends.org/ReadCounter. A more elaborate description is included on the website. To define the gene regions, the Homo_sapiens.GRCh37.75.gtf annotation file was used which is based on the human genome assembly 37 (41). For normalization, the expression per gene/transcript was divided by the combined expression of all genes/transcripts per sample (note that reads that do not map to genes are excluded from the normalization procedure). The resulting data were used to construct the co-expression maps.

To create our co-expression maps, we employed the same approach that coXPRESDB used to construct their microarray-based co-expression map (31). For each possible gene pair combination, a weighted Pearson correlation, based on sample redundancy, was calculated. The sample redundancy is calculated based on the number of similar samples in the dataset, and the sample similarity is measured by the correlation between samples (http://coxpresdb.hgc.jp/help/coex_cal.shtml) (30). Next, a mutual rank was calculated based on the ranking of each gene with its partner. The mutual rank is the average rank of gene A to gene B and gene B to gene A. This causes genes, such as ribosomal genes, that are strongly co-expressed with many other genes to have a lower ranking. This is preferred since these genes are often not of interest for functional enrichment analysis or candidate gene prioritization.

## DATABASE CONTENT AND USER GUIDE

The GeneFriends database, constructed from RNA-seq data, contains co-expression data for 44 248 human genes and for 114 936 transcripts. Transcripts/genes that were not expressed (expression < 10 reads) in at least 10% of the samples were excluded from the co-expression map. As a result, 19 430 out of 63 678 genes and 100 234 out of 215 170 transcripts were excluded. A list of the types of genes found in the co-expression map are shown in Table 1.

**Table 1.** List of genes present in the co-expression maps

	Genes	Transcripts
protein_coding	18658	82528
pseudogene	9483	9888
lincRNA	4997	6221
antisense	4537	6476
miRNA	1024	1017
snRNA	819	814
snoRNA	444	448

A more detailed list can be found in Supplements 9.

**Figure 2.**
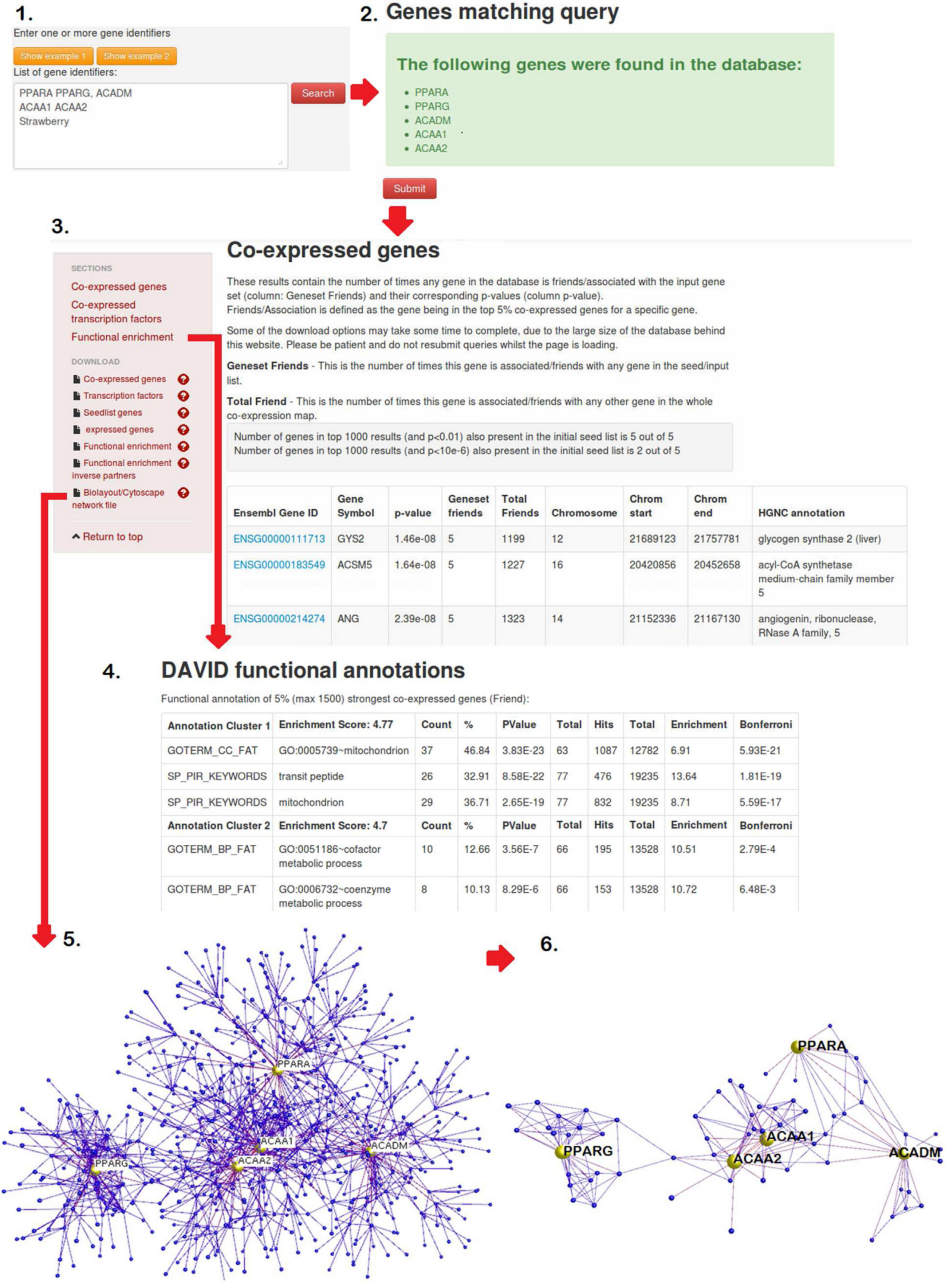
A graphical overview of the steps involved in retrieving results from GeneFriends: (**1**) Insert genes (**2**) Validate input (**3**) Retrieve co-expressed genes (**4**) Investigate functional enrichment (**5**) Visualize network of co-expressed genes using Bioloayout (**6**) Use Biololayout to select the network of interest selecting different thresholds.

To employ GeneFriends the user can submit one or multiple gene/transcript IDs. The results then contain the following sections: (i) a list of the 50 strongest co-expressed genes and the corresponding HGNC annotation for each gene; (ii) a list of the 25 strongest co-expressed transcription factors; (iii) top 20 functional enrichment categories of the co-expressed list of genes, including GO (42), KEGG (43) and OMIM (44). To assess functional enrichment among the co-expressed genes, DAVID web services (45) are used, which is a commonly used tool to assess overrepresentation of functional categories among a list of genes. To obtain the DAVID web results the top 1500 co-expressed genes/transcripts are used (or fewer if there are fewer genes significantly co-expressed (cutoff *P*-value < 10^−6^; since correction for multiple testing using the Bonferroni correction: 0.05/44248 = 1.12*10^−6^ (13)). Additionally, full lists can be downloaded, as well as a network file that can be imported into Biolayout (46) or Cytoscape (47) for visualization and further analyses. Lastly, there is an option to download the functional enrichment of those genes that have an expression pattern which negatively correlates with the expression of the gene(s) of interest, thus those genes with an opposing expression pattern. This is especially interesting for genes/RNAs that downregulate expression of others. Further details can be found on http://www.GeneFriends.org/RNAseq/about/. A graphical overview of the steps involved in retrieving results from GeneFriends is depicted in Figure 2.

## GENE CO-EXPRESSION VALIDATION

To validate our co-expression map and determine the expected false positive rate associated with the calculated Pearson correlations, we scrambled the data and reconstructed the co-expression map. In a previous work we used the top 5% co-expressed genes to investigate functional enrichment as opposed to a co-expression strength cut-off to avoid biases against genes with lower co-expression strengths. To be consistent we used the same approach in this work. To inspect the expected false positive rate using this cut-off, we assumed the values in the co-expression map created from scrambled data as negatives, representing those values that associate with genes that are not co-expressed. The co-expression values present in the co-expression map created from the true data represent the positives. From this we constructed an ROC curve (Supplements 2) and calculated that at a sensitivity of 95% the expected false discovery rate is 4.4%, which occurs at a Pearson correlation > 0.12. The ROC space was 98.3% suggesting that in the top 5% there is a clear distinction between the false positives and the true positives (Supplements 2).

One of the two main purposes of GeneFriends is that users can input a poorly annotated gene or transcript and utilize the functional enrichment of co-expressed genes to associate it with specific biological processes. To validate this approach, we tested nine genes for which the functions are well established. We previously used this approach to validate our microarray-based co-expression map (13) and decided to use the same set of genes. We initially picked three categories: cell cycle, immune system and fatty acid metabolism and picked three genes which we expected co-expressed partners to be functionally enriched for these categories based on known associations. As a result we postulated the following genes to associate with these categories; the cell cycle: *CDC6*, *CDC7*, *CDCA8*; the immune system: *IL10*, *CD4*, *CD8*; fatty acid metabolism: *ACADM*, *PPARA*, *ACAA2* (Supplements 3). We used DAVID (45,48) to identify functional enrichment among the top 5% co-expressed genes. For all genes this showed significant enrichment for the predicted categories, supporting the notion that this approach can be used to elucidate which processes poorly annotated genes play a role in. Moreover, for some genes the more specific roles, such as mitochondrial oxidation for *ACADM* and *ACAA2* within these general processes, showed the strongest enrichment, which are annotated to these specific categories (42). Others such as *PPARA*, that are known to be associated with a wider range of processes (49,50), showed enrichment also for these other processes, underlining the potential of this approach. From these results, we conclude that co-expression results obtained from GeneFriends can be used to predict the processes the genes/transcripts are associated with.

We also compared the co-expressed gene lists from the RNA-seq-based co-expression map to our previously constructed microarray-based map (13). Unlike the RNA-seq-based map, the microarray version was created using a vote counting approach and includes a wider range of data with data from over 4000 experiments rather than the 240 included in the construction of the RNA-seq version. Although these 240 studies describe a wide range of conditions, certain conditions might be overrepresented in sample numbers. We counted the prevalence of terms in the summaries of each sample (Supplements 1) and used Wordl, an application to visualize the prevalence of words in text (http://www.wordle.net/) (Supplements 4). We found the most prevalent terms are ‘stem’ and ‘lymphoblastoid’ which were present in 723/4133 (17.6%) and 716/4133 (17.3%) sample summaries, respectively. There was no strong over representation for any disease related terms with ‘cancer’ (259 samples) being the most prevalent. Since co-expression data have been reported to be tissue- and condition-dependant (14,51), we anticipate differences between microarray- and RNA-seq-based maps. Although the expression ratios of the microarray version cannot be directly compared to the Pearson correlation or mutual rank calculated for the RNA-seq version, it is still possible to compare the ranking of each gene to one another. Only genes present in both co-expression maps were included in this analysis. Doing so for the nine genes described above showed an average overlap of 27% (stdev 9%) of the top 5% co-expressed genes in the microarray with the top 5% co-expressed genes in the RNA-seq version (Table 2). This supports the notion that the co-expression maps are dependent on the data they are constructed from. Nevertheless either co-expression map proves effective at detecting the correct functional enrichment for the nine annotated genes, suggesting that the different co-expressed genes associate with the same or similar functional categories.

**Table 2.** Overlap of the microarray-based co-expression compared to the RNA-seq-based co-expression

	Top 5% vs Top 5%
ACAA2	24%
ACADM	24%
CD4	39%
CD8A	34%
CDC6	31%
CDC7	31%
CDCA8	25%
PPARA	9%
IL10	17%

A more elaborate table can be found in Supplements 10.

## ncRNA VALIDATION

To investigate if it is possible to use GeneFriends to postulate the function of non-coding RNAs, we investigated the functional enrichment of genes co-expressed with 3 annotated non-coding RNAs. One non-coding RNA, EVF-2, known to cooperate with DLx2 which plays a critical role in neuronal differentiation and migration as well as craniofacial and limb patterning during development (52) and two lincRNAs: XIST, a lincRNA active during embryogenesis and associated with X-chromosome inactivation (53) and HOTAIR, a lincRNA that is required for silencing of HOXD genes which if absent causes severe limb and genital abnormalities (54,55).

We found that genes co-expressed with EVF-2 (ENSG00000231764) are strongly enriched for synaptic transmission (1.61E-50) and neuron projection (1.71E-44) (Supplements 5), which is in accordance with our expectations (Bonferroni corrected *P*-values are marked in brackets). XIST was enriched for embryogenic morphogenesis (1.75E-3) and was most strongly enriched for co-expression genes that are involved in transcription (9.10E-57), cell cycle (1.70E-18), chromosome organization (2.33E-21) and zinc finger regions (5.72E-35) (Supplements 5), which are terms we would expect to observe during embryogenesis. We found the co-expressed genes for HOTAIR were enriched for the HOX homeodomain (2.37E-3) and are most strongly enriched for the functional term spermatogenesis (1.72E-13) and reproduction (1.94E-16) (Supplements 5). These results support the notion that GeneFriends can be used to predict functions of ncRNAs.

Since we were curious if functional enrichment could also be detected for genes for which no functional annotation is yet available we also randomly selected poorly annotated genes until we found three with significant functional enrichment. As a result we tested four genes and found significant enrichment for functional categories for three of these genes (Supplements 6), supporting the notion that GeneFriends can assign putative roles to these poorly-studied genes. The following functional enrichment was found for these three genes, which are all genes that have been associated with the lncRNA class (Bonferroni corrected *P*-values marked in brackets): ENSG00000271947, synapse (3.23E-29); ENSG00000258776, visual perception (4.22E-69); ENSG00000232862, sexual reproduction (5.19E-21).

## TRANSCRIPT-SPECIFIC CO-EXPRESSION

Since one of the benefits of the RNA-seq-based co-expression map is that it also contains transcripts, we investigated if it is possible to differentiate between the function of different transcripts originating from the same gene. To this end we have selected a gene that has multiple transcripts, with different co-expression partners, that are known to be involved in different processes: *MACF1*. This is a protein that binds to actin and microtubules (56) and is important for cell motility (57–59).

We identified the two transcripts with the least overlapping partners, ENST00000360115 and ENST00000482035, which shared only 80 out of the 5747 (top 5%) co-expression partners. We next investigated the functional enrichment for the co-expressed transcripts of the two transcripts originating from the same gene. We found that the functional enrichment shows different categories. The top 5% co-expression partners of the ENST00000360115 transcript showed strong enrichment for the GO terms (Bonferroni corrected *P*-values are marked in bracket) ‘synapse’ (2.46 E-27) and ‘neuron projection’ (3.88E-21) whereas ENST00000482035 partners show strong enrichment for ‘regulation of cell motion’ (8.19E-12) and ‘extracellular matrix’ (7.82E-14). The top categories that ENST00000360115 was associated with were not present in the enrichment results for ENST00000482035 and vice versa (Supplements 7). This shows that there can be a clear distinction between the co-expression results obtained from different transcripts originating from the same gene and that it is possible to postulate which genes encode transcripts that lead to proteins involved in different processes.

Next we aimed to identify how often transcripts originating from the same gene are co-expressed with different transcripts. Doing so for each gene) resulted in 294 829 comparisons. Of these 294 829 comparisons, 123 650 have less than 10% of overlapping transcripts in the top 5% co-expressed transcripts. This suggests that different transcripts arising from the same gene are often expressed under different conditions and are likely to play roles in different processes or maybe some are non functional transcripts.

## GENE SET CO-EXPRESSION

The second purpose of GeneFriends (13) is that users can submit a list of genes or transcripts associated with a specific disease or biological process to find other genes/transcripts associated with it. This is particularly of interest with the RNA-seq-based co-expression map as it contains non-coding genes which may play crucial roles in understanding the mechanisms underlying these diseases/processes.

Similar to our previous analysis (13), we used a set of causative cancer genes (60) and identified genes co-expressed with this list of genes. Interestingly, this included a number of genes that one would not find in any microarray-based co-expression map. Using this approach 83 pseudogenes, one microRNA (microRNA 4444–1) and two antisense RNAs (EMC3-AS1, UBL7-AS1) were associated with the cancer seed list (Supplements 8). Genes co-expressed with microRNA 4444–1 (Supplements 8) are strongly enriched for genes involved in transcription (Bonferroni corrected *P*-value: 8.67E-20) and chromatin organization (Bonferroni corrected *P*-value: 2.58E-14), suggesting this microRNA may exert a role in cancer by affecting the expression profile in cancer cells. This is an example of how GeneFriends can be used to associate non-coding factors with diseases/biological processes and how it can help elucidate possible roles of poorly annotated factors uncovered trough this procedure.

## RNA-SEQ-RELATED BIASES

While GeneFriends provides a unique opportunity to elucidate the roles of unstudied genes, it is important to mention a few possible biases that might be present in the RNA-seq co-expression map. Since the co-expression map is created from RNA-seq data, any biases existing in this type of analysis will propagate to the co-expression map, in particular:

- In the library preparation of RNA-seq experiments there is a bias against smaller RNAs (61) for which reason measurements for shorter RNAs such as microRNAs may be less accurate.
- One important step in RNA-seq analysis is to assign reads to genes based on their coordinates. However, in some cases genes overlap with each other, making it hard to assess from which gene the read originates. As a result the read is then ignored. This means that genes that are fully overlapped by other genes can never show expression and becomes a major issue when mapping to transcripts rather than genes as they commonly overlap each other. For this reason we considered ambiguously overlapping reads to represent the expression of each transcript it overlaps with rather than ignoring it. This will mean that transcripts spawning from the same gene are much more likely to show strong co-expression, which is to be considered when retrieving transcript co-expression results from GeneFriends.
- We observed a bias toward positive correlation as opposed to negative correlation. This may be due to the biological nature of the data as negative correlation as a result of negative transcriptional regulation is expected to be much rarer than positive correlation as genes involved in the same biological processes more often co-operate rather than inhibit each other. However, it is not unreasonable to state that the normalization procedure has not yet been optimized for RNA-seq data and normalizing by total read counts has been reported to introduce biases (62). The most commonly applied correction in the past few years calculates FPKM/RPKM values, which correct for gene length. However, these have been extensively debated (62) and new metrics have been suggested (63), which have also been challenged (64). Since none of these normalization protocols have been proven to be perfect, we opted to normalize samples by the total expression of all genes (reads that do not map to genes are excluded as these are more likely to introduce biases), until one of these metrics becomes generally accepted, at which point we will reconstruct the co-expression maps. We are, however, confident that these biases minimally affect the results of our tool, since our aforementioned validation tests have proven consistent with the literature.

## CONCLUDING REMARKS

Over the past century research has led to a better understanding of many diseases and biological processes, however the underlying mechanisms often remain unclear. In research there is a tendency to focus on genes that have already been studied to a broader extent and ignore poorly annotated genes. Yet, it is reasonable to assume that some of the unstudied genes play a crucial role and that without studying them we might never be able to fully understand these diseases and processes. GeneFriends allows researchers to quickly identify poorly annotated genes that are associated with genes that have already been associated with the disease/process under study. This unveils new venues for research and helps uncover new findings as shown for example in (13). This is particularly interesting since GeneFriends also allows association of non-coding RNAs such as microRNAs and lincRNAs. These RNAs have been indicated to play crucial regulatory roles in multiple studies (65–67). Additionally, it is not uncommon that unannotated genes are present in the results of a study, yet since no knowledge is available, they tend to be ignored. GeneFriends can help identify possible roles of these genes which will help experimental design.

Since Next-Generation Sequencing (NGS) is an emerging technology, our proposed RNA-seq co-expression tool will be useful for a growing number of researchers to gather clues regarding the many poorly studied transcripts detected by this approach. Unstudied transcripts or genes differentially expressed in a given RNA-seq analysis can be input into GeneFriends to assess the functional enrichment of co-expressed genes, effectively assigning a putative role to the query transcript/gene and identifying possible interaction partners. Knowing the potential roles of these transcripts will allow the assessment of the most interesting transcripts among those differentially expressed in the process under study and generate hypothesis for testing. This addresses an unmet need for the bioscience community and will help drive post-genome science. GeneFriends is freely available from http://www.GeneFriends.org.

## SUPPLEMENTARY DATA

Supplementary Data are available at NAR Online.
